# Temporary Workers’ Skipping of Meals and Eating Alone in South Korea: The Korean National Health and Nutrition Examination Survey for 2013–2016

**DOI:** 10.3390/ijerph16132319

**Published:** 2019-06-30

**Authors:** Ji-Sook Kong, Kyoung-Bok Min, Jin-Young Min

**Affiliations:** 1 Department of Preventive Medicine, College of Medicine, Hanyang University, Seoul 04763, Korea; 2 Department of Preventive Medicine, College of Medicine, Seoul National University, Seoul 03080, Korea; 3 Institute of Health and Environment, Seoul National University, Seoul 08826, Korea

**Keywords:** temporary worker, eating behaviors, meal skipping, eating alone, Korea

## Abstract

Available evidence suggests that social disadvantages are inextricably linked to unhealthy eating behaviors. Given that temporary workers face insecure employment and uncertainty in their work’s terms and conditions, issues relevant to maintaining healthy eating behavior are likely to be affected. This study investigated the association between temporary employments and, specifically, the status and frequency of meal skipping and of eating alone among temporary and permanent Korean workers. We used data from the 2013–2016 Korean National Health and Nutrition Examination Survey. A total of 5912 working people were included as the study population. We classified them as temporary workers (*n* = 3036) and permanent workers (*n* = 2876). Eating behaviors included meal skipping and eating alone. The rate and frequency of meal skipping and eating alone were higher in temporary workers. After adjustment for potential confounders, the likelihoods for temporary workers’ skipping lunch was twice as high (OR = 1.95, 95% CI 1.45–2.63) as for permanent workers. In particular, temporary workers had four-fold-increased odds (OR = 4.12, 95% CI 2.29–7.41) of eating alone three times per day relative to permanent workers. We found that temporary workers were more likely to skip meals and eat alone than were permanent workers.

## 1. Introduction

Over the past three decades, globalization, financial crises, and the global economy’s restructuring have promoted labor market ‘flexibility’ and entailed changes to work arrangements, especially in terms of the growth of temporary employment [1]. Temporary employment can be defined as wage employment relations other than those of unlimited duration, including fixed-term, subcontracted jobs, project or task-based contracts, as well as seasonal or casual work [2]. Although temporary jobs encompass a wide and diverse range of occupations and sectors, they tend to offer low wages and diverse poor working conditions (e.g., less access to paid vacations, sick leave, and unemployment insurance) relative to permanent jobs [3]. These detrimental aspects of temporary employment result in psychological morbidity and socioeconomic disadvantages for temporary workers [4,5].

Many studies have noted that temporary workers are more likely than standard workers to have poor health outcomes (e.g., cardiovascular disease, depression, anxiety, and suicide) [6,7,8], experience absenteeism caused by work-related injury or sickness [9], and choose unhealthy lifestyles (e.g., heavy smoking and alcohol dependence) [10]. In addition, temporary workers are more likely to have more difficulties accessing healthcare [11] and developing friendships in the workplace than are standard workers.

Eating is essential for survival, and healthy eating is essential for health and well-being [12,13,14]. Eating behavior is affected by individual perception (of health and nutrition benefits and of body weight) as well as the objective realities of socio-economic environments and physical access [15,16]. For example, lower education and lower occupation have been associated with fewer intakes of certain food products (i.e., vegetables, fish, and vitamins) [17], and skipping breakfast with low family socioeconomic status and sedentary lifestyle [18]. Employed parents’ good working conditions positively affected the eating of more homemade meals, eating with the family, and less meal skipping, while employed parents’ work conditions (e.g., long hours) were associated with missed breakfast, use of convenience entrees, and more restaurant meals [19]. The available evidence suggests that social disadvantages are inextricably linked to unhealthy eating behaviors. Since temporary workers face insecure employment and uncertainty in the terms and conditions of their work [20,21], all issues relevant to maintaining healthy eating behavior could be affected. However, not much is known about working arrangements’ (i.e., contract types) effects on eating behaviors.

Therefore, the present study examined the association between temporary employments and eating behavior. Specifically, we compared the status and frequency of meal skipping and eating alone between temporary and permanent Korean adult workers.

## 2. Materials and Methods

### 2.1. Study Population

We used data from the 2013–2016 Korean National Health and Nutrition Examination Survey (KNHANES) conducted by the Centers for Disease Control and Prevention [22]. The KNHANES, was designed to assess Koreans’ health and nutritional status by extracting representative samples via multistage, probability-cluster, and complex sampling design. The overall participation was 77.5% for the 4-year study period (KNHANES 2013–2016; 31,098 of 40,127). Of those 31,098 participants, we selected a population aged 19–64 years (*n* = 12,823). We initially included 7603 after excluding cases that were missing information on whether a wage worker was a permanent worker or not (*n* = 10,672). Among those, we excluded participants who did not complete questionnaire items on eating habits (*n* = 1113) such as meal skipping, meal frequency per week, and eating alone, or who were under dietary control (*n* = 578), for example starving or fasting for reasons such as disease or weight loss. Thus, the final sample was 5912 working people. All of the participants provided written informed consent. Also, because the KNHANES constitutes a publicly opened national statistical database without personally identifiable information, we performed a secondary analysis of the data without IRB approval.

### 2.2. Variable Definition

A waged worker is someone who is employed and paid by another person or company and has worked for more than one hour to earn pay for the past week (except for the self-employed). Identification of contract type was based on the following question: “What is your current job status?”. Possible responses were “permanent job (guaranteed employment until retirement)” and “temporary job”, according to which, respondents were classified as permanent workers and temporary workers, respectively.

Eating behaviors included meal skipping and eating alone. Skipping of breakfast, lunch, or dinner was determined by whether the respondent had eaten each meal on the day prior to the survey, via “yes or no” questions. Frequency of meal skipping was based on the question, “During the past year, how many days a week did you eat breakfast, lunch, or dinner?” Response included 5–7/week, 3–4/week, 1–2/week, and almost never. Frequencies were categorized into two groups: ≥5/week and <5/week. For eating alone, participants were asked, “When you have been eating breakfast, lunch, and dinner for the past year, have you usually eaten with someone else?” Responses were “yes” or “no.” Participants also were asked to indicate how often they ate their daily meals (i.e., breakfast, lunch, or dinner) alone, as once, twice, or three times. Other variables included socio-demographic characteristics and health behaviors and conditions. The self-reported socio-demographic variables were age (19–29, 30–39, 40–49, or 50–64), gender, marital status (married, divorced/widowed, or never), number of family members (1 or ≥2), and household income (1st quartile, 2nd quartile, 3rd quartile, or 4th quartile). Jobs were classified into white collar (managerial, professional, or clerical), pink collar (services or sales), and blue collar (manual labor). Health behaviors included cigarette smoking (never, former, or current) and alcohol drinking (yes or no). Body mass index (BMI) was categorized into underweight (0 < BMI <18.5), normal (18.5 ≤ BMI < 25), and overweight/obesity (BMI ≥ 25.0).

### 2.3. Statistical Analysis

Statistical differences in general characteristics between temporary and permanent workers were analyzed using the chi-square test. We compared eating behaviors such as meal skipping, meal frequency per week, and frequency of eating alone per day between temporary and permanent workers. To assess the association between temporary employment and eating behavior, we designated permanent workers as a reference group and conducted unadjusted and adjusted logistic regression models. The result provided odds ratios (OR) of temporary employment with 95% confidence intervals (CI). In the regression models, Model 1 was adjusted for demographic characteristics (i.e., age, gender, marital status, number of family members (1 or ≥2), and household income); Model 2 was adjusted for Model 1 + job types; and Model 3 was adjusted for Model 2 + health behavior and condition (i.e., cigarette smoking, alcohol drinking, and BMI). The KNHANES data was based on a complex survey design, survey non-response and post-stratification to represent the civilian, non-institutionalized Korean population. The sample weights were estimated by the inverse of selection probabilities and inverse of response rates by adjusting them to the sex- and age-specific Korean populations [23]. All of the statistics were based on sampling weights in order to take complex sampling into account and were performed with SAS 9.4 software (SAS Institute, Cary, NC, USA). Statistical significance was set at *p* ≤ 0.05.

## 3. Results

### 3.1. General Characteristics of Study Population

Of the 5912 participants, 2876 workers were permanent and 3036 workers were temporary. Table 1 displays the general characteristics of the study’s population, revealing significant differences in all demographic variables, health behaviors, and health conditions between the groups. Temporary workers were more likely to be younger or older, to be female, to be divorced/widowed, to live in one-person households, to have low income, and to be engaged in pink- or blue-collar jobs than permanent workers. They were more likely to be never smokers, non-drinkers, and underweight than permanent workers.

### 3.2. Comparisons of Eating Behaviors Between Temporary and Permanent Workers

**Figure 1:**
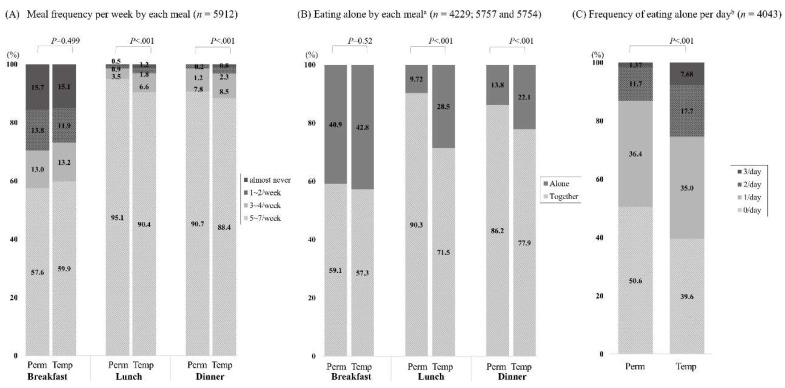
Percentage of eating behaviors by permanent and temporary worker status (2013–2016). Results were reported as unweighted percentages and the *p*-value was calculated based on the complex sample design and weights. Perm: permanent worker; Temp: temporary worker. ^a^ Frequency of eating alone was evaluated in participants who had three or more meals per week (breakfast, lunch, and dinner, respectively). ^b^ Frequency of eating alone was evaluated in participants who had three or more meals per day (breakfast, lunch, and dinner).

We compared eating behaviors between the temporary and permanent workers (Table 2 and Figure 1). Except for breakfast, eating behaviors for each meal differed significantly between those groups. Temporary workers had relatively higher rates of meal skipping (lunch 7.52 vs. 5.50%; dinner 6.43 vs. 4.59%) and lower rates of meal frequency per week (≥5/week; lunch 90.4 vs. 95.1%; dinner 88.4 vs. 90.7%). The rate of eating alone was significantly higher in temporary workers than in their permanent counterparts (28.5 vs. 9.72% for lunch and 22.1 vs. 13.8% for dinner). The frequency of eating alone was higher in temporary workers: eating alone once per day, 60.4 vs. 49.4%; eating alone for two meals per day, 25.4 vs. 13.0%, and eating alone for three meals per day, 7.68 vs. 1.37%. 

### 3.3. Association Between Temporary Employment and Eating Behaviors

Table 3 displays the ORs (95% CI) for temporary workers’ eating behaviors. In the unadjusted regression model, temporary workers had a higher likelihood of skipping meals and eating alone, except for breakfast, than permanent workers. We then included confounding variables (i.e., age, sex, marital status, number of family members, household income, job type, cigarette smoking, alcohol drinking, and BMI) and gradually adjusted them in regression models 1–3. The adjusted ORs for skipping breakfast, lunch, and dinner were no longer significant in temporary workers relative to permanent workers. On the other hand, the likelihood of eating lunch less than five times a week was significantly higher in temporary workers than in permanent workers after adjustment for potential confounders (Model 3; OR = 1.95, 95% CI 1.45–2.63). The adjusted ORs for temporary workers’ eating alone were significantly higher than for permanent workers, specifically, eating lunch alone (OR = 2.77, 95% CI 2.30–3.34), eating dinner alone (OR = 1.25, 95% CI 1.04–1.51), eating alone once per day (OR = 1.39, 95% CI 1.18–1.64), twice per day (OR = 1.67, 95% CI 1.33–2.11), and three times per day (OR = 4.12, 95% CI 2.29–7.41). We analyzed models 1–3 by adding confounders to the crude model. As has been reported of an association between demographic variables and dietary behavior, such as between meal skipping and eating alone [24,25], we found that the addition of demographic variables (age, sex, marital status, number of family members) to the crude model had the highest impact (odds ratio) on skipping meals and eating alone as covariates. In model 2, the variable of job type was added to model 1 and, in model 3, the variables of health behavior and conditions were added to model 2. The difference of result (odds ratio) between model 2 and model 3 was small.

## 4. Discussion

Using nationally representative sample data on South Koreans, this study examined whether temporary employment was associated with unhealthy eating behaviors. We found that temporary workers were more likely to skip lunch and eat alone than were permanent workers. The likelihood for temporary workers’ skipping lunch was twice as high as for permanent workers. Eating alone was predominant among temporary workers and, particularly, their odds for eating alone three times per day were four-fold increased relative to permanent workers. Thus, our findings support prior evidence of poor eating behaviors among socially disadvantaged groups [26,27]; furthermore, our findings suggest that temporary employment contributes to inequalities in healthy eating behaviors.

Interest in the eating behaviors of skipping meals and eating alone has increased, because unhealthy behaviors alter the quantity and quality of food consumed, thereby affecting health outcomes [28]. People who skipped a meal were more likely to consume foods containing high level of cholesterol and carbohydrates and to have lower intakes of fruits, vegetables, vitamins, and minerals than those who never skipped a meal [29,30]. Further, those who skipped a meal faced increased risks to their cardio-metabolic health, notably of obesity and diabetes [31,32]. Additionally, eating alone has been associated with reduced energy intake [33], food diversity according to assessment of dietary quality using the 11-item scale [34], and inadequate intake through social interaction influence during mealtime [35]. In this way, eating alone can lead to detrimental effects on physical (metabolic syndrome) and mental health (depressive symptoms), even among those who live with family members [23,24].

Previous studies have focused on temporary workers’ inferior health status [4,21]. The association between temporary employment and health is complex, though it has been explained by unhealthy behaviors (e.g., smoking, excess alcohol use, and sedentary leisure activity) in response to flexible employment [10]. In this context, temporary workers’ unhealthy eating behaviors are not likely to differ significantly.

To our knowledge, this study is the first to show a significant association between temporary employment and unhealthy eating behaviors, i.e., skipping lunch and eating alone. Little evidence has been found on the effects of work types on eating behaviors [36]. Nevertheless, our findings are supported by prior results on the potential link between poor working conditions (e.g., job stress, long working hours, and shift work) and unhealthy diets [19]. In a study of Japanese male workers, negative psychological responses induced by job stress (e.g., fatigue, tension/anxiety, and depression) were significantly associated with eating behaviors causing obesity, namely with substitute eating and drinking and feelings of satiety, as well as motivations for eating [37]. In addition, observational studies have shown that workers engaged in rotating shift work exhibited more unbalanced diets (e.g., high fat intake and low intake of vegetables) and abnormal temporal eating patterns (e.g., skipping breakfast and/or late dinner) [38,39].

Based on the above research, the observed association between temporary employment and unhealthy eating behaviors is complex. Employment characteristics—specifically, greater job uncertainty, lower income, more limited workplace rights and social protection, and greater imbalance of power between employers and workers than in standard employment—could shape eating practices. Future studies are needed to confirm our findings and to examine potential mechanisms.

This study has several limitations. First, because of its cross-sectional design, we could not establish causal links between temporary employment and unhealthy eating habits. Confirming an association between them might require a prospective study or a longitudinal study, in which changes of employment status are accompanied by certain eating behaviors. Second, although this study was based on national representative data for the general population, our study sample was not truly representative of working people, due to possible bias arising from missing data on job status (contract type) and eating habits and due also to the exclusion of participants who were under dietary control (e.g., starving or fasting for reasons of disease or weight loss). However, this lack of representativeness was unlikely to have affected the association we observed between temporary employments and eating behaviors. Third, in the KNHANES survey, eating behaviors and confounding variables were investigated through retrospective, self-reports; thus, there remains a potential for recall and nonresponse biases. Finally, although we included many confounding variables including demographics and health behaviors, the effects of unmeasured confounders (e.g., job stress and work schedule) were not fully controlled in the statistical model.

## 5. Conclusions

In conclusion, we found that in Korea’s working population, temporary workers skipped more meals and ate alone more than permanent workers. Despite this study’s mentioned limitations, it provides preliminary evidence on unhealthy eating behaviors associated with contract type. Efforts to encourage temporary workers’ healthy eating behaviors might be important in reducing health inequality due to employment status.

## Figures and Tables

**Table 1:** Characteristics of permanent and temporary workers (*n* = 5912).

Variables	Total (*n* = 5912) ^1^	Permanent (*n* = 2876)	Temporary Workers (*n* = 3036)	*p*-Value ^2^
Age (years)				
19–29	948 (16.0)	349 (12.1)	599 (19.7)	<0.0001
30–39	1479 (25.0)	956 (33.2)	523 (17.2)	
40–49	1659 (28.1)	921 (32.0)	738 (24.3)	
50–64	1826 (30.9)	650 (22.6)	1176 (38.7)	
Gender				
Male	2893 (48.9)	1779 (61.9)	1114 (36.7)	<0.0001
Female	3019 (51.1)	1097 (38.1)	1922 (63.3)	
Marital status				
Married	4202 (71.2)	2258 (78.6)	1944 (64.1)	<0.0001
Divorced/widowed	411 (6.96)	90 (3.13)	321 (10.6)	
Never	1292 (21.9)	524 (18.3)	768 (25.3)	
Number of family members				
Single	416 (7.04)	147 (5.11)	269 (8.86)	<0.0001
≥two members	5496 (93.0)	2729 (94.9)	2767 (91.1)	
Household income				
1st quartile	373 (6.32)	51 (1.78)	322 (10.6)	<0.0001
2nd quartile	1337 (22.7)	435 (15.2)	902 (29.8)	
3rd quartile	1968 (33.4)	943 (32.9)	1025 (33.8)	
4th quartile	2222 (37.7)	1441 (50.2)	781 (25.8)	
Job type				
White-collar	2883 (49.0)	1867 (65.4)	1016 (33.5)	<0.0001
Pink-collar	1070 (18.2)	305 (10.7)	765 (25.2)	
Blue-collar	1937 (32.9)	685 (24.0)	1252 (41.3)	
Cigarette smoking				
Never	3388 (57.5)	1439 (50.1)	1949 (64.5)	<0.0001
Former	1129 (19.2)	677 (23.6)	452 (15.0)	
Current	1375 (23.3)	754 (26.3)	621 (20.6)	
Alcohol drinking				
Drinker	3769 (64.0)	1982 (69.1)	1787 (59.1)	<0.0001
Non-drinker	2123 (36.0)	888 (30.9)	1235 (40.9)	
Body weight status				
Underweight	284 (4.83)	118 (4.13)	166 (5.48)	0.0002
Normal	3804 (64.7)	1847 (64.7)	1957 (64.6)	
Obesity	1795 (30.5)	890 (31.2)	905 (29.9)	

^1^ number (%). ^2^ *p*-value was calculated based on complex sample design and weights.

**Table 2:** Eating behaviors of participants by permanent and temporary worker status (*n* = 5912).

Variables		Total (*n* = 5912) ^1^	Permanent (*n* = 2876)	Temporary Workers (*n* = 3036)	*p*-Value ^2^
Meal skipping (on previous day)					
Skipping breakfast	Yes	1479 (25.0)	713 (24.8)	766 (25.3)	0.1132
	No	4429 (75.0)	2162 (75.2)	2267 (74.7)	
Skipping lunch	Yes	386 (6.53)	158 (5.50)	228 (7.52)	0.0002
	No	5522 (93.5)	2717 (94.5)	2805 (92.5)	
Skipping dinner	Yes	327 (5.53)	132 (4.59)	195 (6.43)	0.0021
	No	5581 (94.5)	2743 (95.4)	2838 (93.6)	
Meal frequency per week (in previous 1 year)					
Breakfast frequency per week	<5/week	2430 (41.3)	1218 (42.5)	1212 (40.2)	0.6898
	≥5/week	3458 (58.7)	1651 (57.6)	1807 (59.9)	
Lunch frequency per week	<5/week	432 (7.34)	142 (4.95)	290 (9.61)	<0.0001
	≥5/week	5456 (92.7)	2727 (95.1)	2729 (90.4)	
Dinner frequency per week	<5/week	615 (10.4)	266 (9.27)	349 (11.6)	0.0003
	≥5/week	5273 (89.6)	2603 (90.7)	2670 (88.4)	
Eating alone (in previous 1 year)					
Eating breakfast alone	Yes	1770 (41.9)	827 (40.9)	943 (42.8)	0.5183
	No	2459 (58.2)	1196 (59.1)	1263 (57.3)	
Eating lunch alone	Yes	1111 (19.3)	275 (9.72)	836 (28.5)	<0.0001
	No	4646 (80.7)	2553 (90.3)	2093 (71.5)	
Eating dinner alone	Yes	1037 (18.0)	389 (13.8)	648 (22.1)	<0.0001
	No	4717 (82.0)	2438 (86.2)	2279 (77.9)	
Frequency of eating alone per day (*n* = 4043) ^3^					
Eating alone once or more per day	Yes	2225 (55.0)	975 (49.4)	1250 (60.4)	<0.0001
	No	1818 (45.0)	998 (50.6)	820 (39.6)	
Eating alone two or more times	Yes	783 (19.4)	257 (13.0)	526 (25.4)	<0.0001
	No	3260 (80.6)	1716 (87.0)	1544 (74.6)	
Eating alone three times per day	Yes	186 (4.60)	27 (1.37)	159 (7.68)	<0.0001
	No	3857 (95.4)	1946 (98.6)	1911 (92.3)	

^1^ Results were reported as unweighted percentages (%). ^2^ *p*-value was calculated based on complex sample design and weights. ^3^ Frequency of eating alone was evaluated for participants who had three or more meals per day.

**Table 3:** Odds ratios (95% CI) of eating behaviors for temporary workers (*n* = 5912).

Models	Permanent	Unadjusted	Model 1	Model 2	Model 3
Skipping breakfast (on previous day), yes	ref (1)	1.11 (0.98–1.25)	1.04 (0.90–1.21)	1.00 (0.85–1.17)	0.99 (0.85–1.16)
Skipping lunch (on previous day), yes	ref (1)	1.54 (1.22–1.95)	1.41 (1.09–1.82)	1.28 (0.98–1.67)	1.25 (0.96–1.64)
Skipping dinner (on previous day), yes	ref (1)	1.48 (1.15–1.91)	1.09 (0.83–1.43)	1.03 (0.78–1.35)	1.02 (0.78–1.34)
Breakfast frequency per week (in previous 1 year), <5/week	ref (1)	1.02 (0.91–1.15)	1.07 (0.92–1.23)	1.06 (0.91–1.23)	1.05 (0.91–1.23)
Lunch frequency per week (in previous 1 year), <5/week	ref (1)	2.29 (1.80–2.93)	2.07 (1.56–2.74)	1.93 (1.44–2.60)	1.95 (1.45–2.63)
Dinner frequency per week (in previous 1 year), <5/week	ref (1)	1.43 (1.18–1.74)	1.07 (0.87–1.32)	1.01 (0.81–1.26)	1.00 (0.81–1.25)
Eating breakfast alone (in previous 1 year), yes	ref (1)	1.05 (0.91–1.21)	1.00 (0.85–1.17)	1.02 (0.86–1.20)	1.03 (0.87–1.22)
Eating lunch alone (in previous 1 year), yes	ref (1)	3.65 (3.10–4.30)	2.88 (2.40–3.46)	2.76 (2.29–3.33)	2.77 (2.30–3.34)
Eating dinner alone (in previous 1 year), yes	ref (1)	1.73 (1.48–2.03)	1.24 (1.03–1.48)	1.24 (1.03–1.50)	1.25 (1.04–1.51)
Eating alone once or more per day (*n* = 4043), yes ^1^	ref (1)	1.48 (1.28–1.71)	1.35 (1.15–1.59)	1.37 (1.16–1.61)	1.39 (1.18–1.64)
Eating alone two or more times (*n* = 4043), yes ^1^	ref (1)	2.19 (1.81–2.65)	1.70 (1.36–2.11)	1.68 (1.34–2.11)	1.67 (1.33–2.11)
Eating alone three times per day (*n* = 4043), yes ^1^	ref (1)	6.07 (3.73–9.87)	4.19 (2.37–7.42)	4.17 (2.32–7.52)	4.12 (2.29–7.41)

Model 1 (demographics) was adjusted for demographic characteristics (i.e., age, sex, marital status, number of family members (1 or ≥2) and household income); Model 2 was adjusted for Model 1 + job type; Model 3 was adjusted for Model 2 + health behavior and condition (i.e., cigarette smoking, alcohol drinking, and BMI). ^1^ Frequency of eating alone was evaluated in participants who had three or more meals per day.
